# Advances in Synthetic Strategies for Microalgal Carotenoid Enhancement and Emerging Applications

**DOI:** 10.3390/antiox15030359

**Published:** 2026-03-12

**Authors:** Peipei Xu, Yurong Wang, Chunli Luo, Anqi Xue, Hong Du, Jing Chen

**Affiliations:** 1 Guangdong Provincial Key Laboratory of Marine Biotechnology, STU-UNIVPM Joint Algal Research Center, Institute of Marine Sciences, Shantou University, Shantou 515063, China; 2 Guangdong Engineering Technology Research Center of Offshore Environmental Pollution Control, Shantou University, Shantou 515063, China; 3 Guangdong Provincial Key Laboratory of Marine Disaster Prediction and Prevention, Shantou University, Shantou 515063, China

**Keywords:** microalgae, carotenoid synthesis, improvement pathways, emerging applications

## Abstract

Carotenoids are increasingly studied for their robust antioxidant capacity, anti-inflammatory potential, protective vision and validated contribution to human health. Carotenoids are mainly obtained through chemical synthesis and plant extraction, which results in relatively high costs for producing carotenoids. However, microalgae represent a sustainable and high-yield platform for natural carotenoid production, with advantages including rapid growth, high pigment accumulation, and broad environmental adaptability. This review summarizes recent biotechnological advances in enhancing carotenoid production, with a focus on metabolic engineering, environmental regulation, and cultivation strategies. CRISPR/Cas9 enables precision metabolic pathway engineering, while environmental factors like light, nutrients, and stress significantly influence yield. Different cultivation strategies allow carotenoids to fulfill commercial or research needs. The two-stage strategy achieves rapid biomass increase during the growth stage, then shifts to accumulate carotenoids. This regulatory mode significantly reduces cell death by continuous stress, providing high productivity and stability in large-scale production. Carotenoids participate in many innovative applications across various fields, including treatments in medicine, skin protection in cosmetics, protein stabilization in foods, enhancing animals’ survival and so on. Future research will integrate bioprocess optimization, precision strain engineering, and adaptive environmental strategies to scale high-value microalgal carotenoid production as a commercially and environmentally viable solution.

## 1. Introduction

Carotenoids are red, orange, and yellow pigments naturally synthesized by plants and microorganisms [1]. To date, over 1117 different natural carotenoids from 683 source organisms have been identified, exhibiting diverse structures and biological functions [2,3]. They perform important physiological functions such as antioxidant, tissue-restorative, anti-inflammatory, and anticancer activities [4]. The studies demonstrated the role of carotenoids in preventing and mitigating serious health disorders [5]. They also function as UV-protective agents shielding the skin and other organs from the harmful effects of solar UV radiation [6].

From food and skincare products to pharmaceutical applications, carotenoids are widely utilized as feed additives in aquaculture and as natural food colorants. However, many commercial carotenoids are predominantly synthetic, which raises concerns about their potential health risks relative to natural alternatives. These concerns have driven increasing global demand for natural carotenoids [7]. Microalgae, as single-cell organisms, have recently garnered significant interest worldwide due to their valuable metabolites (e.g., carotenoids, lipids, proteins, pigments, and polysaccharides) [8]. These microorganisms serve as promising bioproduction platforms due to their rapid growth, facile cultivation and harvesting, waste stream valorization capability, and high-yield synthesis of valuable bioproducts [9]. Their ecological roles contribute to biochemical diversity and phylogenetic significance [10].

**Figure 1:**
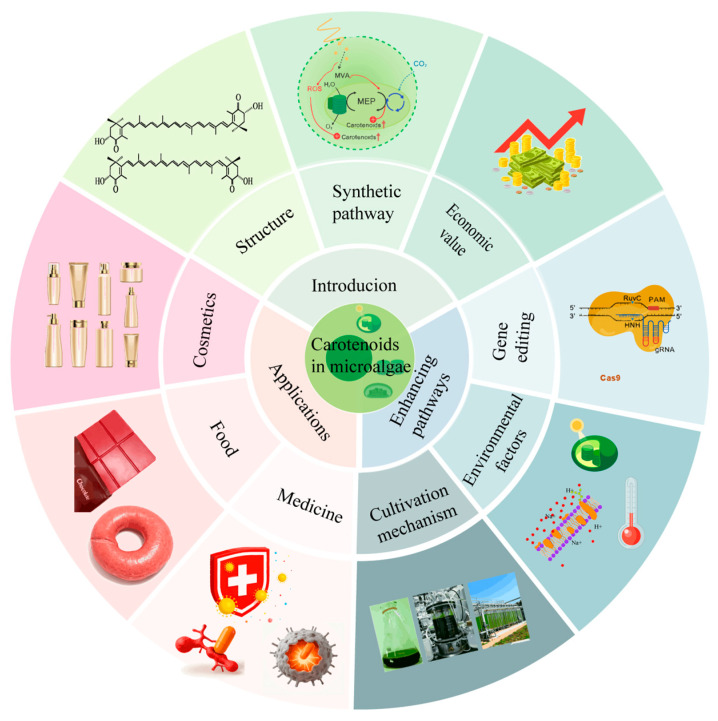
Introduction, enhancement pathways and emerging applications of microalgal carotenoids.

The previous reviews have extensively discussed the extraction and downstream processing of microalgae as critical components of bioprocessing [11]. They highlighted the antioxidant capacity of microalgal carotenoids and explored biotechnological, engineering, and downstream approaches [12]. However, with the development of emerging technologies, strategies for enhancing carotenoids in microalgae are evolving, yet a systematic synthesis of these advancements remains lacking. Therefore, this review focuses on analyzing the economic feasibility of major production platforms, the construction of engineered high-yielding strains, and the challenges they face in large-scale production. The core innovation of this review focuses on the synthetic strategies for enhancing microalgal carotenoids and their emerging applications, such as the engineered *Chlorella zofingiensis* and its market potential. It also systematically summarizes the current application status of microalgal carotenoids. Finally, this paper not only aims to integrate information but also endeavors to point out future research directions and promote more promising avenues (Figure 1).

## 2. Carotenoids in Microalgae

**Figure 2:**
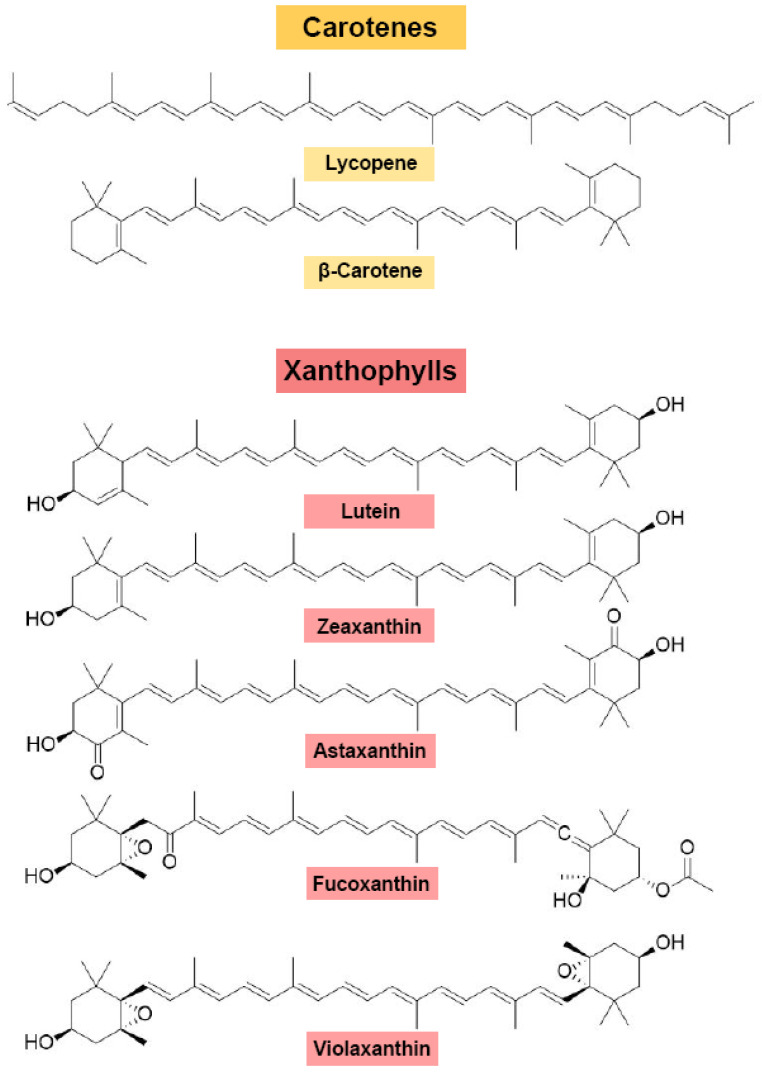
Structures of predominant carotenoids biosynthesized in microalgae. All xanthophyll structures contain hydroxyl groups and six-membered rings. Additionally, astaxanthin and fucoxanthin contain carbonyl groups [32].

Carotenoids, a class of isoprenoid lipids, are the most widely distributed and structurally diverse natural pigments [1]. And it is characterized as C40 lipophilic tetraterpenes with at least ten conjugated double bonds (Figure 1) [13]. Structurally, they are divided into two primary classes: carotenes, which are hydrocarbons, and xanthophylls, which contain oxygen functional groups (Figure 2) [14]. With over 700 identified variants, these compounds play crucial biological roles. Also, their antioxidant activity helps prevent UV-induced free radical damage, inhibit lipid peroxidation, and reduce harmful molecule production [15]. Additionally, carotenoids enhance cell membrane stability by facilitating the absorption, harvesting, and transfer of light energy. Among them, the α-, β-, and γ-carotene serve as vitamin A precursors [16]. While several microalgae produce valuable carotenoids, their commercial viability is highly species-dependent and influenced by specific cultivation challenges. Critically evaluating the major carotenoids found in microalgae, we focus not only on their structural diversity and functional significance but also on the biotechnological potential and economic feasibility of their natural production.

### 2.1. Astaxanthin from Microalgae: A Comparative Analysis

Astaxanthin (C_40_H_52_O_4_) is a potent ketocarotenoid characterized by keto and hydroxyl moieties on both ionone rings (Figure 2). It is renowned for its exceptional antioxidant capacity, demonstrating radical-quenching activity 10-fold greater than β-carotene and 500-fold higher than α-tocopherol, earning it the designation as “super vitamin E” [17]. The commercial production of astaxanthin from microalgae presents a trade-off between yield, production scalability, and economic feasibility, with primary producers being green algae (Chlorophyta).

*Haematococcus pluvialis* (Chlorophyta) stands as the primary industrial choice for natural astaxanthin production. Its dominant commercial status is underpinned by an exceptional capacity to accumulate over 50 mg/g (5% of dry weight) of astaxanthin under stress conditions [18]. Its principal advantage lies in its extremely high accumulation in cells, which has established it as the yield standard and enabled its widespread application in aquaculture, health foods, and pharmaceuticals [19,20]. However, the commercial scalability of *Haematococcus pluvialis* faces significant bottlenecks, including a slow growth rate and high susceptibility to contamination. Furthermore, it requires complex and costly two-stage cultivation systems, involving a growth stage followed by stress induction, that result in high energy consumption.

*Chlorella zofingiensis* (Chlorophyta) emerges as a promising engineered platform for astaxanthin production. By employing advanced strategies such as glucose supplementation, nitrogen deprivation, and high-light induction, it has achieved unprecedented astaxanthin yields of up to 36.79 mg/L [21,22]. Its key comparative advantages include a faster growth rate than *Haematococcus pluvialis* and high potential for process optimization and genetic engineering. However, its industrial adoption is currently hindered by lower cellular accumulation and the need for significant genetic modification to achieve commercial competitiveness.

*Nannochloropsis* spp., in which astaxanthin production is first documented among Eustigmatophytes, have been utilized as a platform for producing carotenoids (Table 1). While its reported astaxanthin yield is notably lower than green algae (Chlorophyta) and making it non-competitive as a primary source—its key advantage lies in its valuable co-product profile [23]. It concurrently produces β-carotene, fucoxanthin, lipids and vitamin E [24]. This multi-product output may improve overall process economics through a biorefinery approach. Consequently, its commercial viability for astaxanthin production likely depends on the market value and recovery efficiency of these co-products rather than only on astaxanthin.

The future of astaxanthin from microalgae-derived production does not lie in finding a “perfect” species. In short, the choice of microalgae platform is not simply about pursuing the highest yield, but rather a strategic trade-off among production cost, technological complexity, and product diversification.

### 2.2. β-Carotene from Microalgae: Industry Success and Limitations

β-carotene, a yellowish-orange tetraterpenoid (C_40_H_56_) (Figure 2), is a critical provitamin A source with diverse biological functions. It supports eye health by aiding vision pigment production to prevent night blindness and cataracts, while also reducing liver toxin buildup and boosting immune function [32]. The microalgae industry’s first success in β-carotene production is the green alga *Dunaliella salina*.

*Dunaliella salina* stands as the premier β-carotene production platform, renowned for its extraordinary capacity to accumulate over 14% of its dry weight under stress conditions [33,34]. This unparalleled productivity has cemented its commercial strengths. The economic significance of β-carotene is substantial; when combined with astaxanthin, these two pigments alone account for up to 35% of global carotenoid revenue, underscoring its immense market value [35]. Despite the commercial success of *Dunaliella salina*, the production of β-carotene from microalgae faces significant inherent limitations that impact its economic feasibility. The high yields in *Dunaliella salina* and similar species are achieved through the application of stressful environmental conditions (e.g., high salinity, light intensity, or nutritional deficiency) [33]. A major limitation is that these stress strategies significantly increase the consumption of energy, elevating production costs and environmental impact [23,36]. While high-yield strains exist, there is a fundamental trade-off between maximizing cellular β-carotene content and achieving high biomass productivity at an industrial scale. Optimizing this balance remains a critical challenge for producers. Although β-carotene is distributed throughout many groups of microalgae, research and commercial exploration have heavily focused on a few species like *D. salina* [14]. The potential of other microalgae, such as *Nannochloropsis* spp., which also produce β-carotene alongside other valuable compounds [25]. It remains underexplored, potentially missing opportunities for more sustainable or cost-effective production systems.

In conclusion, the microalgae-based β-carotene industry presents a sample of successful biotechnology commercialization, single-handedly led by the remarkable biological capacity of *Dunaliella salina*. However, this success is tempered by persistent challenges related to the high energy costs of production and the biological trade-offs of stress-induced synthesis. Future advancements will focus on overcoming these limitations through genetic engineering of high-yield strains to reduce reliance on costly stress induction and the development of more efficient photobioreactor designs. Additionally, the exploration of integrated biorefinery approaches, where β-carotene is one valuable product among others, improves overall process economics.

### 2.3. Lutein and Other Xanthophylls from Microalgae: Emerging Sources and Challenges

Lutein (C_40_H_56_O_2_) (Figure 2), a dihydroxy xanthophyll derived from α-carotene, is a critical dietary carotenoid with profound importance for human health. It also acts as a critical ocular pigment in humans, reducing light-induced retinal damage by 40% [37]. Moreover, its chromatic expression is dose-dependent, exhibiting yellow coloration at low concentrations and red-orange at higher concentrations [38]. Microalgae represent a highly promising and sustainable platform for lutein production, with several species showing significant commercial potential. Species within the genus *Chlorella vulgaris* (Chlorophyta) are well-established and commonly used for commercial-scale production of lutein, alongside other carotenoids [23,36]. Notably, the strain *Asterarcys quadricellulare* (Chlorophyta) has been identified as a particularly high-yield producer, capable of synthesizing up to 28.7 mg/g of lutein (in combination with β-carotene), highlighting its potential as a superior production platform [39]. Moreover, some red algae are showing significant commercial potential to produce lutein. The economically valuable species *Pyropia yezoensis* has been characterized, identifying lutein, zeaxanthin, α-carotene, and β-carotene as major carotenoids in both thallus and conchocelis (Table 1). Research has also identified lutein-5,6-epoxide and key monohydroxy intermediates for lutein synthesis in this species, offering novel insights into its biosynthetic pathways in red seaweeds [40].

Despite the identified potential, the path to commercializing microalgal-derived lutein faces several significant challenges. A major challenge across production systems is the substantial energy consumption associated with the stress-induction strategies. These strategies are required to trigger enhanced lutein accumulation in many species. This significantly elevates operational costs and negatively impacts overall economic feasibility, a noted bottleneck in green algae production [23]. For promising non-traditional sources like red algae (e.g., *Pyropia yezoensis*), there is a significant lack of in-depth research on the specific action mechanisms, regulation, and efficiency of lutein biosynthesis across different growth stages. While *Chlorella* is commercially established, the development and utilization of lutein from other highly promising sources (e.g., *Pyropia yezoensis*) remain in the preliminary stages. Processes for efficient extraction, purification, and scale-up for these emerging sources are not yet mature, creating a bottleneck for commercial translation [41].

There is a pressing need for the application of advanced genetic engineering and metabolic engineering to develop robust, high-yielding strains that accumulate lutein without the need for costly stress induction. Concurrently, innovations in bioreactor design and cultivation processes are essential to reduce production costs and achieve true commercial competitiveness against microalgae-based sources.

### 2.4. Carotenogenesis Pathways

**Figure 3:**
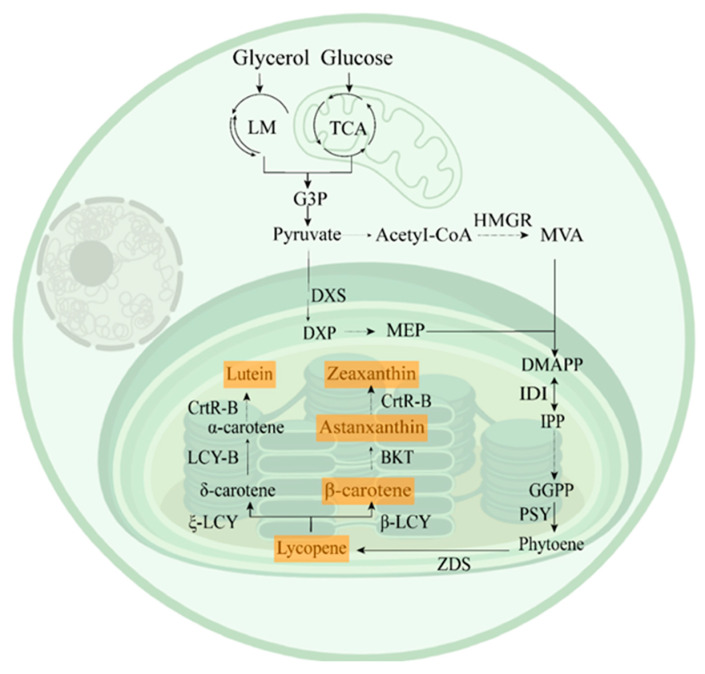
The carotenoid biosynthetic pathway in microalgae. The MVA pathway and lipid metabolism take place in the cytoplasm, the TCA cycle happens in the mitochondria, and the others occur in the chloroplasts. LM, Lipid metabolism; TCA, Tricarboxylic acid cycle; DXP, 1-Deoxy-D-xylulose-5-phosphate; DXS, 1-Deoxy-D-xylulose-5-phosphate synthase; MEP, Methylerythritol 4-phosphate; Acetyl-CoA, Acetyl coenzyme A; HMGR, 3-Hydroxy-3-methylglutaryl-CoA reductase; MVA, Mevalonate; DMAPP, Dimethylallyl pyrophosphate; IDI, Isopentenyl-diphosphate delta-isomerase; IPP, Isopentenyl pyrophosphate; GGPP, Geranylgeranyl pyrophosphate; PSY, Phytoene synthase; ZDS, Z-carotene desaturase; β-LCY, β-Lycopene cyclase; ζ-LCY, ζ-Lycopene cyclase; BKT, β-carotene ketolase; LCY-B, Lycopene cyclase-B; CrtR-B, Carotenoid β-ring hydroxylase [44,46].

The carotenogenesis pathways differ across species. Carotenoid biosynthesis in microalgae is a highly conserved process that originates from two C_5_ precursors: isopentenyl diphosphate (IPP) and dimethylallyl diphosphate (DMAPP). These precursors are primarily synthesized via the plastid-localized methylerythritol 4-phosphate (MEP) pathway, with the cytosolic mevalonate (MVA) pathway operating in some species (Figure 3) [41,42,43].

The committed step begins with the condensation of IPP and DMAPP to form geranylgeranyl diphosphate (GGPP). The first rate-limiting reaction is catalyzed by phytoene synthase (PSY), which condenses two GGPP molecules to form phytoene [44]. Then phytoene sequentially desaturates by phytoene desaturase (PDS) and ζ-carotene desaturase (ZDS), yielding lycopene. Lycopene undergoes cyclization at the ends of the molecule: lycopene ε-cyclase (ε-LCY) introduces an ε-ring to form α-carotene, while lycopene β-cyclase (β-LCY) introduces a β-ring to yield β-carotene (Figure 3). Further modifications, including hydroxylation, epoxidation, ketolation, and glycosylation, diversify the carotenoid profile, leading to the formation of valuable carotenoids such as astaxanthin, lutein, and fucoxanthin [45].

The carotenoid biosynthetic pathway in microalgae represents a complex, regulated network that integrates primary metabolism with specialized secondary metabolism (Figure 3). This integration confers significant economic value across multiple sectors, including metabolic engineering, pharmaceuticals, food, cosmetics and so on.

## 3. The Economic Value of Carotenoids

**Figure 4:**
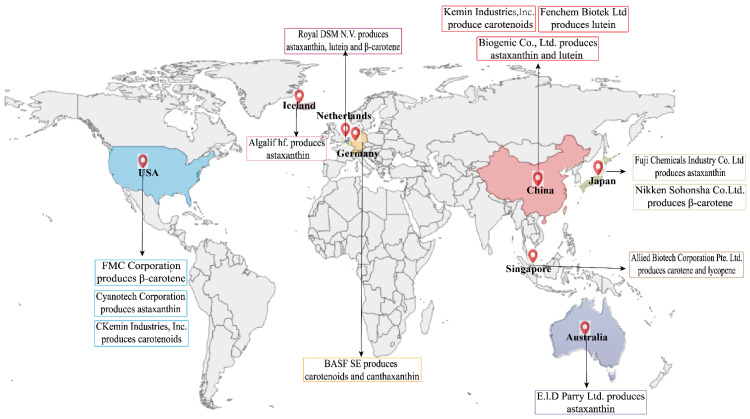
The companies produce different kinds of carotenoids and their locations. The companies which in the same country produce different types of carotenoids by using the same color, such as USA (blue) [54,55,56], Iceland (pink) [57], Netherlands (purple) [58], Germany (yellow) [59], Singapore (brown) [60], China (red) [61,62], Japan (green) and Australia (dark blue) [9,63]. The professional terms involved: Inc., Incorporated; Ltd., Limited; Co., Company.

Carotenoids have high economic value due to their unique structures that enhance bioactivity and increase production costs, making them widely used in natural food colorants, nutraceuticals, and feed additives [47]. The high cost is driven by unique structural features of carotenoids, including a characteristic keto functionality at the 4 or 4′ position on the β-ionone ring and potential hydroxyl groups at the 3 and 3′ positions [3,47]. These features confer significant antioxidant activity, resulting in their widespread use in medicinal applications and animal feed supplements [48]. Surveys forecast a lutein market value of 357.7 million U.S. dollars by 2024 and a global market of carotenoids of $2.5 billion by 2028 (Figure 1) [2]. The geographical distribution of companies engaged in global carotenoid production and indicated the compound is distributed in many countries and most of them are concentrated in relatively developed economic regions, such as the United States, Europe, and Southeast Asia (Figure 4). Nowadays, the overall carotenoid market was valued at $1.8 billion, with β-carotene prices ranging from 300 to 3000 per kg, will reach $2.8 billion by 2030, growing at a compound annual growth rate (CAGR) of 5.2% [48]. Ketocarotenoids, especially astaxanthin, are one of the most expensive carotenoids on the market, with astaxanthin commanding prices ranging from $2500 to $10,000 per kg. The market value of nutraceutical-grade astaxanthin is estimated at more than $6000 per kg. The global astaxanthin market was valued at approximately US $1.5 billion in 2022 and is projected to expand at a compound annual growth rate (CAGR) of ~6.5% to 7.5%, reaching US $2.4–2.8 billion by 2028–2030 [49]. The share of carotenoids in the global market is expanding at an accelerated pace, especially in the food, pharmaceutical applications, and aquaculture feed sectors. In the future, their industrial status will be upgraded from auxiliary additives to fundamental health elements [50].

Carotenoids possess significant economic value as themselves and their derivatives participate in health-promoting biological activities that contribute to reducing risks of several diseases, including various cancers (e.g., breast, colorectal, gastric, oral), heart disorders (e.g., atherosclerosis), and ocular diseases [51,52,53]. Recent research further reveals their potential benefits for cognitive performance, Parkinson’s disease, respiratory conditions, diabetes mellitus, obesity, metabolic disorders, and perinatal health [14]. This extensive therapeutic profile underscores the significant economic potential of carotenoids, particularly in pharmaceutical and nutraceutical applications. Although carotenoids have important economic value, their production is concentrated in developed regions. These may be related to the cost pressure they face and the bottleneck of biosynthesis. Currently, only a limited number of countries have achieved industrial-scale production. Subsequently, the cost can be reduced by modifying the key genes for carotenoid synthesis to achieve efficient expression. Additionally, utilizing high-value by-products generated in the microalgae production process further contributes to cost reduction.

## 4. Carotenoid Pathway Engineering in Microalgae

Metabolic engineering has been widely applied to enhance carotenoid production in green microalgae through genome editing and enzyme modification. Beyond genetic and pathway manipulation, environmental factors and culture media composition have also been exploited to elevate carotenoid yields. The feasibility of using CRISPR/Cas9 to knock out genes and enhance carotenoid production has been successfully demonstrated [64]. To increase astaxanthin and canthaxanthin production, the pigment content can be boosted under environmental stresses such as elevated light and/or salt stress.

### 4.1. Gene Editing Technology

The genome editing technology, particularly the CRISPR/Cas9 system, facilitated the metabolic engineering of carotenoid biosynthesis in microalgae (Figure 1). CRISPR/Cas9 is a promising genome editing tool for enhancing carotenoid production, enabling directed evolution of metabolic pathways via gene knockouts, knock-ins, and multiplex editing. Overexpression or activation of *PSY* based on CRISPR/Cas9 in *Haematococcus pluvialis* elevated the amount of astaxanthin produced by 300% under stress, identifying this rate-setting enzymatic activity as a master regulator [65]. In *Dunaliella salina*, CRISPR/Cas9-mediated disruption of both lycopene ε-cyclase (ε-LCY) and zeaxanthin epoxidase (ZEP) increased zeaxanthin accumulation by 60% [66]. In *Chlorella vulgaris*, co-expression of GGPP synthase (GGPPS) and β-LCY increased β-carotene fivefold, demonstrating that the combination of strategies can enhance metabolic flux toward the target product [67]. These studies illustrate that targeted editing of genes related to carotenoid synthesis through CRISPR/Cas9 technology can significantly increase the production of carotenoids.

However, several challenges persist in the metabolic engineering of microalgae, including persistent off-target mutation risks. Multiplex genome editing introduces complexity in guide RNA design, delivery efficiency, and expression balancing, hindering its scalability and reproducibility. Keeping metabolic balance during high carotenoid production is critical to prevent overconsumption of essential nutrients or imbalances that slow growth or trigger stress [1].

### 4.2. Environmental Factors

At the postgenomic level, carotenoid synthesis in microalgae is influenced by the environment and metabolism (Figure 1). The overproduction of ROS (Reactive Oxygen Species) under high irradiance activates ROS-dependent MAPK (Mitogen-Activated Protein Kinase) cascades, up-regulating b-ZIP transcription factors and carotenogenic genes (e.g., *PSY*, *LCYB*) [68]. In *Haematococcus pluvialis*, exposure to blue light significantly enhanced astaxanthin content, upregulated *BKT* gene expression by 11.7-fold [69]. Nitrogen starvation regulated the TOR kinase to relieve repression on the carotenoid gene clusters and activate the biosynthesis of isoprenoids [70]. Under nitrogen starvation, the levels of β-carotene in *Dunaliella salina* increased up to 14% of DW, accompanied by a decrease in the glycerol/carotenoid from 5.2 to 0.8 [49]. *Dunaliella salina* cultivated at 2.5 mol/L NaCl produced 8.7 mg/L β-carotene, accompanied by a 3-fold glycerol accumulation [71]. Temperature changes also influence the efficiency of carotenoid synthesis, primarily β-carotene. The high temperatures (30–35 °C) facilitate the heat-shock factor (HSF) that binds to the *PSY* promoter, enhancing phytoene synthase expression and increasing carotenoid yield by 18.3% [72].

Although the synthesis of microalgal carotenoids can be effectively enhanced by regulating environmental signals (e.g., light, temperature, salinity) and inducing metabolic changes (e.g., nitrogen starvation), this strategy still faces dual limitations. Maintaining specific stress environments (e.g., high intensity, high salt concentration, or temperature control conditions) requires continuous energy input, significantly increasing production costs. The inherent environmental adaptability of microalgae causes the expression and activity of key carotenoid synthesis enzymes (e.g., PSY, LCYB) to revert to baseline when the stress is removed, with the product accumulation rate returning to levels at or below the pre-induction baseline [73,74].

### 4.3. Cultivation Mechanism

A bioreactor can maintain the target metabolic state of microalgae for a long time by controlling the environment. Currently, there are many types of systems used, including photobioreactors, heterotrophic fermentation, mixotrophic cultivation systems, two-stage cultivation, and so on (Figure 1).

Photoautotrophic cultivation utilizes light energy and CO_2_ as the carbon substrate for precise environmental control. Elevating light intensity during *Dunaliella salina* cultivation enhances β-carotene accumulation to 17.7 mg/g dry weight, mediated by light-induced upregulation of carotenoid biosynthetic enzymes [75]. Heterotrophic fermentation relies on organic carbon assimilation to achieve high-cell-density production, exemplified by *Chlorella zofingiensis* yielding 12.5 mg/L astaxanthin under 30 g/L glucose supplementation [76]. Mixotrophic cultivation integrates photosynthetic and organic carbon assimilation, *Dunaliella salina* producing 32.0 mg/L β-carotene through bicarbonate-boosted photo-mixotrophy [77]. Another method is two-stage cultivation, which decouples biomass growth from product accumulation. In *Haematococcus pluvialis*, nutrient-replete growth is followed by nitrogen deprivation combined with high irradiance, triggering astaxanthin accumulation to 30 mg/g dry weight—a fourfold increase compared to single-stage cultivation [78]. Collectively, trophic modes and staged stress applications establish a strong technical foundation for scaled-up high-titer microalgal carotenoid production.

## 5. Emerging Application of Carotenoids in Microalgae

Currently, with the improvement of the economic value and cultivation technology of microalgal carotenoids, the applications of microalgal carotenoids are expanding, involved in the medicine, food, cosmetic and other industries.

### 5.1. Medicine of Microalgal Carotenoids

(1) Antioxidant mechanisms of microalgal carotenoids

Microalgal carotenoids have demonstrated strong antioxidant activity in various experimental models, suggesting their potential to mediate a multi-layer defensive system at the cellular and systemic level. These pigments, including astaxanthin, fucoxanthin, lutein, and zeaxanthin, scavenge reactive oxygen species (ROS) [79,80]. In preclinical studies, astaxanthin derived from *Haematococcus pluvialis* was shown to scavenge superoxide, hydroxyl, and peroxyl radicals, reducing lipid peroxidation by 60–80% in rat liver slices [81]. Similarly, an animal study showed that fucoxanthin from *Odontella aurita* restored liver levels of antioxidant enzymes (SOD, Superoxide Dismutase) to normal in diabetic rats [82]. In addition, in vitro evidence indicates that lutein and zeaxanthin from *Euglena gracilis* effectively protect against harmful light-activated oxygen produced by certain antibiotics, providing strong light protection in human blood [83]. Importantly, a clinical trial demonstrated that Astaxanthin significantly ameliorated drug-induced hepatotoxicity, reducing ALT (Alanine Aminotransferase) by 38% and MDA by 29% while boosting total antioxidant capacity by 24% in clinical trials [84]. Collectively, these preclinical and clinical findings suggest that microalgal carotenoids may hold broad application prospects in adjuvant treatment for diseases such as nephrotoxicity, hepatotoxicity, and metabolic syndromes.

(2) Anti-inflammatory actions of microalgal carotenoids

Astaxanthin inhibits the phosphorylation of IκBα and prevents the NF-κB complex from moving into the nucleus, thereby reducing inflammatory gene transcription in UVB-irradiated human skin cells [59]. In clinical trials, astaxanthin supplementation reduced some blood inflammation markers in metabolic syndrome patients [85]. Additionally, lutein and zeaxanthin, abundant in *Chlorella vulgaris*, have been found to suppress inflammation in retinal pigment epithelial cells by blocking ROS-triggered inflammasome activation [86]. In a mouse model, dietary fucoxanthin (50–100 mg/kg) prevented DSS-induced colon inflammation in mice by strengthening the gut barrier [87]. These studies indicated that the carotenoids in microalgae are gradually being applied in reducing inflammation or in anti-inflammatory treatment with other drugs. Despite promising results, the clinical development of microalgal carotenoids as anti-inflammatory agents faces hurdles. The effective concentrations used in vitro (e.g., 50 μM for NF-κB inhibition) are difficult to achieve in human tissues through diet or standard supplementation. Future work may exploit pharmacokinetics and validate effects as standalone or adjunct therapies in well-defined inflammatory diseases through clinical trials.

(3) Anticancer potential of microalgal carotenoids

Microalgal carotenoids have demonstrated significant anticancer activities. For instance, in vitro studies show that treatment with 10–50 μM astaxanthin can trigger G2/M arrest and mitochondrial apoptosis in liver cancer cells, which can be used for the adjuvant treatment of liver cancer cells (Table 2) [88]. Similarly, fucoxanthin inhibits human colon cancer cells (HCT-116) through ROS-mediated p38 MAPK activation and intrinsic apoptosis [89]. A synergistic effect was observed in breast cancer cells (MCF-7), lutein and β-carotene were found to suppress cell growth by inhibiting PI3K/Akt/mTOR signaling and enhancing chemosensitivity to doxorubicin [90]. In hamsters, astaxanthin supplementation suppresses DMBA-induced oral carcinogenesis by inhibiting angiogenesis and suppressing the inflammatory microenvironment [91]. Furthermore, clinical epidemiological data indicate an inverse correlation between dietary lutein/zeaxanthin intake and colorectal cancer [92]. These findings substantiate the potential of microalgal carotenoids as multifunctional agents in cancer prevention and treatment. Nevertheless, despite encouraging evidence, establishing a direct causal relationship and advancing therapeutic applications requires deeper mechanistic investigation.

### 5.2. Food in Microalgal Carotenoids

Microalgae-derived carotenoids, as natural pigments, are widely used in functional food ingredients. Currently, carotenoids dominating both research and commercial markets include β-carotene, astaxanthin, lutein and zeaxanthin [36,50]. β-carotene, mainly produced by *Dunaliella salina* under nitrogen-limiting stress and high-light, achieves accumulations up to 14% DW [33]. It is commercially utilized as a provitamin A supplement and a natural colorant, with successful applications in functional foods such as ricotta cheese and beverages [100]. Astaxanthin, widely recognized as one of the most potent natural antioxidants, is accumulated by *Haematococcus pluvialis* at concentrations exceeding 50 mg/g dry weight. Its exceptionally high antioxidant activity and associated health benefits command a high market value. However, production is technologically challenging and capital-intensive, typically requiring a two-stage process in closed photobioreactors to control contamination and optimize yield. These factors constrain scalability and increase production costs. Its deep-red pigment serves as a bioactive ingredient in products such as cookies and white chocolate, enhancing both protein stability and antioxidant capacity (Figure 1) [101,102,103]. Lutein, regarded as a primary xanthophyll, accumulates to 4–5% DW in *Chlorella protothecoides* under heterotrophic fed-batch conditions. When incorporated into bread at 2–6% DW, it imparts a stable golden color and enables lutein production exceeding 60 kg/mg [104,105]. Zeaxanthin, often co-extracted with lutein like *Scenedesmus almeriensis* or *Nannochloropsis oculata*, is utilized as a retina-targeted functional pigment in plant-based milks and nutrition bars [106]. In applications, spray-dried instant soups (0.5–2% DW) have successfully delivered more than 2 mg/g fucoxanthin serving without compromising flavor characteristics [107].

### 5.3. Cosmetics in Microalgal Carotenoids

Microalgal carotenoids are promising bioactive classes for next-generation skincare actives. It offers environmental benefits and provides more benefits than plant extracts or synthetic compounds [63].

Astaxanthin is recognized as the most potent natural singlet oxygen quencher, with activity approximately 100 times greater than vitamin C [108]. Also, astaxanthin inhibits UV-induced peroxidation and suppresses the NF-κB/COX-2 pathway to relieve inflammation and photoaging (Figure 1) [109].

Fucoxanthin extracted from *Phaeodactylum tricornutum* utilizes its unique oxygen-containing groups to target skin pigment enzymes [110]. Moreover, fucoxanthin absorbs both UVA and UVB spectra (λ_max_ 330–450 nm) and quenches lipid radicals, thereby achieving dual photoprotection and whitening functions [108].

With the development of technology, high-energy visible light (HEV) from digital screens can cause periorbital fatigue and increase fine lines. Lutein and zeaxanthin from microalgae can effectively relieve these symptoms. The conjugated both of them can block 30–40% of blue light and inhibit the production of reactive oxygen species in retinal photoreceptor cells, thereby alleviating related fine lines [83].

Additionally, β-carotene from *Dunaliella salina* provides provitamin A activity, promoting keratinocyte renewal and enhancing the epidermal barrier. Experiments have proven that after sun exposure, the use of 0.1% β-carotene can increase the ceramide level in the stratum corneum by 18% and reduce epidermal water loss by 9% [57]. A representative multi-carotenoid formulation has been shown to brighten skin by 1.5 shade grades and reduce squalene peroxides by 35%—effects unattainable with any single carotenoid [111].

The carotenoids from microalgae in the cosmetics field possess powerful antioxidant capabilities, which can effectively resist the damage of free radicals, delay skin aging, and bring long-lasting care to the skin. Thus, carotenoids are expected to make them a critical direction for the emergence of cosmetics research.

### 5.4. Other Potential Applications of Carotenoids in Microalgal

Compared with synthetic pigments, consumers now show a clear preference for healthy, natural colorants. Microalgal astaxanthin is an important nutritive factor capable of boosting the success rate and quality of artificial seed-rearing plans for crustaceans and finfish alike. In aquafeeds, the carotenoids in microalgae improve larval survival, flesh quality, external coloration, and so on. A feeding trial demonstrated that Atlantic salmon receiving 50 mg/kg of astaxanthin from *Haematococcus pluvialis* exhibited a 1.7-fold longer survival time [112]. In *Exopalaemon carinicauda* shrimp, adding natural microalgae astaxanthin to feed boosted growth, survival, and red/yellow coloration, while raising muscle astaxanthin levels. And at 400 mg/kg, tissue astaxanthin surged 17.8-fold with clear weight gain [113]. Similarly, *Paralithodes camtschaticus*, fed a diet containing 380 µg/g of microalgal astaxanthin, showed improvements in survival, growth, and colouration [114].

Microalgae can be deployed for wastewater treatment. A photobioreactor system inoculated with *Scenedesmus* sp. removed over 95% nitrogen and 88% phosphorus from urban effluent, while accumulating intracellular carotenoids up to 2.1% of dry weight [115]. The microalgae-based treatment system achieves water purification, biomass production, and pigment recovery, enabling the extraction of resulting antioxidants as natural colorants. In addition, microalgal carotenoids can be utilized for biodiesel production. Whole-cell incorporation of astaxanthin-rich *Haematococcus pluvialis* biomass into biodiesel synthesis reduces production costs by 18% per kg of astaxanthin-enriched biodiesel, further enhancing economic viability [116].

Building upon these multifaceted advantages, future research should focus on building all-in-one systems that clean dirty water, make valuable colorants, and produce green energy from microalgae, creating a low-carbon, reuse-resource economy.

## 6. Further Perspectives

Microalgal carotenoids demonstrate significant application potential; however, several bottlenecks must be addressed to achieve broader commercialization, enhancing yield through synthetic biology-based strain engineering to decouple pigment synthesis from stress-induced growth inhibition. Additionally, developing economical and environmentally friendly downstream extraction technologies to minimize reliance on organic solvents, and implementing circular biorefinery approaches to valorize biomass residues and repurpose waste streams. Addressing these interdisciplinary challenges is essential to fully realize the potential of microalgal carotenoids as sustainable next-generation health products. These challenges span biotechnology, process engineering, and clinical research.

## 7. Conclusions

This review summarizes recent advances in microalgal carotenoid research to increase carotenoid production. Safety concerns surrounding chemically synthesized carotenoids for human consumption are driving a growing demand for natural alternatives. Carotenoids are high-value natural pigments with strong antioxidant, anti-inflammatory, anticancer, and photoprotective properties. These make them widely applicable in pharmaceuticals, cosmetics, functional foods, and related industries.

Microalgae, characterized by rapid growth, high carotenoid yields, and robust environmental adaptability, represent a promising and sustainable platform for industrial carotenoid production. Microalgal carotenoid yields have been enhanced by CRISPR/Cas9 genome editing, high-efficiency photobioreactors, and two-stage cultivation strategies. Future research will concentrate on boosting efficiency and reducing costs. CRISPR/Cas9 and related high-throughput tools enable accelerated strain development, while deeper insights into the physiology of carotenogenesis will support species-specific process optimization. However, major bottlenecks must be addressed to achieve sustainable and economical large-scale production. These include the high energy consumption and capital costs of large-scale photobioreactors, inefficient and costly downstream extraction processes, and challenges in light and nutrient utilization efficiency during mass cultivation. Despite the challenges in large-scale cultivation and cost control, the commercial prospects for microalgal carotenoids are extremely promising. Continued innovation in bioprocess engineering and biological design will be critical to overcoming bottlenecks and establishing microalgae as a competitive source of natural carotenoids.

## Figures and Tables

**Table 1:** List of the carotenoid-producing microalgae.

Classification	Microorganism	Types of Carotenoids	Concentration	Reference
Rhodophyta	*Madagascaria erythrocladioides*	Carotenoids	2.23 mg/L	[25]
	*Porphyridium aerugineum*	Zeaxanthin	0.4 mg/g	[26]
		β-Carotene	0.4 mg/g	
Chlorophyta	*Haematococcus pluvialis*	Astaxanthin	50 mg/g	[18]
	*Dunaliella salina*	β-Carotene	0.36 mg/L	[27]
	*Chlorella reinhardtii*	Canthaxanthin	0.023 μg/L	[28]
		Neoxanthin	0.58 mg/g	
	*Chlorella vulgaris*	Violaxanthin	0.26 mg/g	[29]
		Lutein	2.18 mg/g	
	*Chlorella sorokiniana*	Astaxanthin	25.1 mg/L	[30]
	*Chlorella zofingiensis*	Canthaxanthin	4.8 mg/L	[22]
		β-Carotene	3.9 mg/L	
		Lutein	4.4 mg/L	
		Violaxanthin	12.5 mg/L	
Eustigmatophyceae	*Nannochloropsis*	Astaxanthin	7.4 mg/L	[24]
		β-Carotene	2.2 mg/L	
		Fucoxanthin	1.1 mg/L	
Bacillariophyceae	*Phaeodactylum tricornutum*	Fucoxanthin	8.0 mg/g	[31]

**Table 2:** Health benefits and mechanisms of carotenoids confirmed by research.

Classified	Health Benefit	Mechanism	Reference
Lycopene	Heart protection	Antiproliferative apoptosis	[93]
β-carotene	Antioxidant defense	Singlet oxygen neutralization	[94]
	Vitamin precursor	Converted to retinol	[95]
Lutein	Cataract prevention	Retinal blue-light filtration	[96]
	Blue-Light antioxidant	Lipid/protein protection	[93]
Astaxanthin	Anti-inflammatory	Inflammatory modulation	[97]
	Cardiovascular health	Vascular enhancement	[98]
Fucoxanthin	Anti-obesity	Lipid gene regulation	[99]
	Anti-inflammatory	Cytokine suppression	[99]

## Data Availability

The data presented in this study are openly available in databases such as Web of Science, Scopus, PubMed, and Google Scholar. All data discussed and analyzed in this review article are obtained from the published studies cited within this manuscript. Complete details of these sources are provided in the reference list.
